# Supplementary material for: Applying machine learning techniques to predict the properties of energetic materials
Source: Sci Rep. 2018 Jun 13;8:9059. doi: 10.1038/s41598-018-27344-x (PMC5998124; doi:10.1038/s41598-018-27344-x)
Supplement: Supplementary file 1 — Supplementary Information [file 41598_2018_27344_MOESM1_ESM.pdf]

Applying machine learning techniques to predict the properties of energetic materials

Daniel C. Elton,[1] Zois Boukouvalas,[1] Mark S. Butrico,[1] Mark D. Fuge,[1] and Peter W. Chung[1]
Department of Mechanical Engineering, University of Maryland, College Park, 20742, United States

(Dated: 24 May 2018)

I. SUPPLEMENTARY METHODS: MACHINE LEARNING BASIC WORKFLOW & TERMINOLOGY

In the machine learning workflow, data is first collected, cleaned, and standardized. The target property values are put into a vector $\boldsymbol{y}$. Next, the raw data $\boldsymbol{x}_i^{\text{raw}}$ is transformed into fixed-length feature vectors $\boldsymbol{x}_i$ that are useful for machine learning, a step called featurization. The feature vectors become the rows in the design matrix $\boldsymbol{X}$. Machine learning algorithms take $\boldsymbol{X}$ and $\boldsymbol{y}$ as inputs and attempt to find a function $f(\boldsymbol{x}) = y^{\text{pred}}$ such that a loss function is minimized. The machine learning model defines a space of functions through which the search is performed. The loss function we use is the squared error loss, defined as :

$$L_{\text{se}}(\{\boldsymbol{x}_i\}, \boldsymbol{y}, f(\boldsymbol{x})) = \sum_{i=1}^{N} (y_i - f(\boldsymbol{x}_i))^2 \quad (1)$$

Additionally, we add a regularization term to the loss function

$$L(\{\boldsymbol{x}_i\}, \boldsymbol{y}, f(\boldsymbol{x})) = L_{\text{se}} + \alpha ||\boldsymbol{w}||^D \quad (2)$$

here $\boldsymbol{w}$ is the "weight vector" containing the tuneable parameters in the model. In what follows we use $D = 2$, corresponding to an L2 norm, except for in LASSO regression, which uses $D = 1$. Regularization penalizes model functions of higher complexity. Regularization makes machine learning models less prone to overfitting (fitting noise in the data) and less sensitive to ill effects from outliers and strongly correlated features. The parameter α, sometimes called the "regularization strength" sets the degree of regularization and is an example of a hyperparameter. Hyperparameters are set in advance before the machine learning model is trained.

II. SUPPLEMENTARY METHODS: STACKING MODELS

We tried a few different variations on the idea of model stacking, a type of ensemble learning.[1,2] In model stacking the outputs of a set of trained models are used as features for another model which makes the final prediction. We experimented with a stacking strategy where a model for density prediction was trained first, and then the output of that model was used as an additional feature when training a model for explosive energy. The improvement in performance was minimal.

III. SUPPLEMENTARY INFORMATION: TRAIN-TEST SPLITTING METHODS & GENERALIZATION PERFORMANCE

In an attempt to reduce the gap between the train and test errors (ie. generalization error or overfitting), we tried several popular train-test spliting strategies (table III). We compared standard shuffle split, stratification over the chemical groups and stratification over clusters. Clusters were determined using k-means clustering with $k = 10$ (10 clusters). Plots of the clustering objective function (essentially a measure of average variance from the cluster centroids) vs the number of clusters used were analyzed. There was no clear "elbow point" indicating an natural number of clusters in the data, so we choose $k = 10$ clusters. No significant differences were observed in the results, although in the case of the Huang & Massa data the gap between train and test r values decreased slightly. We also looked into using "scaffold split" which is implemented in the *deepchem* Python package. Scaffold split groups molecules by their chemical scaffold (backbone). We determined the method would not be very useful to implement as many of the scaffolds appeared only once in the data.

IV. SUPPLEMENTARY INFORMATION: DESCRIPTIONS OF FINGERPRINTS

Atom-Pairs (Carhart et al. 1985)[3] - encodes the list of all atom pairs in the molecule. Each atom pair is a triplet of information consisting of (*atom description i*, *distance ij*, *atom description j*). *distance ij* is the distance between atom i and atom j as measured by the shortest path length along the molecular bond network, and each *atom description* is a specification of the atom type by atomic number, number of π-bonding electrons, and number of hydrogens. Each possible atom pair is encoded in a unique integer. The list of atom pairs becomes a list of integers which can then be transformed into a bit vector of desired length.
Topological Torsions (Ramaswamy et al., 1987)[4] - encodes the list of all topological torsions in the molecule. Each topological torsions is sequence of four bonded non-hydrogen atoms which is encoded in a quartet of information consisting of (*atom description i*, *atom description j*, *atom description k*, *atom description l*). Each *atom description* is an integer containing information on the atomic number and number of π-bonding electrons. As

name	MAE_{train}	MAE_{test}	$MAPE_{test}$	$RMSE_{test}$	R^2_{train}	R^2_{test}	r_{train}	r_{test}
shuffle	0.30 ± 0.01	0.35 ± 0.07	9.87	0.423	0.82	0.63	0.91	0.83
stratified over groups	0.30 ± 0.01	0.38 ± 0.09	11.16	0.454	0.82	0.71	0.91	0.86
stratified over clusters	0.30 ± 0.01	0.36 ± 0.06	9.90	0.435	0.83	0.62	0.91	0.85

TABLE I. Comparison of different splitting techniques for the Huang & Massa data, prediction of explosive energy, using kernel ridge regression and the combined (Estate+Custom Descriptor Set+Sum over Bonds) featurization. The groups were Pyrazole ($N = 20$), Cubane ($N = 12$), HMX ($N = 6$), Linear ($N = 18$), Butterfly ($N = 10$), Ketone ($N = 7$), TNT ($N = 16$), RDX ($N = 6$), Ring ($N = 8$), CL20 ($N = 6$).

name	MAE_{train}	MAE_{test}	$MAPE_{test}$	$RMSE_{test}$	R^2_{train}	R^2_{test}	r_{train}	r_{test}
shuffle	0.15 ± 0.00	0.17 ± 0.03	2.18	0.249	0.89	0.82	0.94	0.91
stratified over groups	0.15 ± 0.00	0.17 ± 0.03	2.20	0.254	0.89	0.81	0.94	0.91
stratified over clusters	0.15 ± 0.00	0.17 ± 0.03	2.16	0.250	0.89	0.82	0.94	0.91

TABLE II. Comparison of different splitting techniques for the sensitivity data, prediction of detonation velocity, using kernel ridge regression and the combined (Estate+Custom Descriptor Set+Sum over Bonds) featurization. The groups were designated Unstable ($N = 21$), NAC ($N = 86$), NNO2 ($N = 77$), CNO2 ($N = 66$), ONO2 ($N = 13$), 5mArN ($N = 45$).

with atom-pair, each resulting type of topological torsion in the molecule is encoded in a unique integer, and a list of integers is generated for further processing.

Extended Connectivity Fingerprints (ECFPs) (Rogers & Hahn, 2010)[5] This fingerprinting method was developed at Accelrys, Inc. in 2000 and is also known as Morgan circular fingerprinting, as it is a efficient version of the classic Morgan fingerprinting algorithm (Morgan, 1965).[6] ECFPs are one of the most popular graph-based fingerprints and encode information about graph substructures. The algorithm for generating an ECFP is as follows:

> An initial assignment stage in which each atom has an integer identifier assigned to it. This initial list of integers is added to the fingerprint set. The iterative updating stage – each atom collects its own identifier and the identifiers of its immediately neighboring atoms into an array. A hash function is applied to reduce this array back into a new, single-integer identifier. This new list of integers is added to the fingerprint set. This process is iterated a specified number of times d, known as the "width" of the fingerprint. The duplicate identifier removal stage - multiple occurrences of the same substructure are reduced to a single representative in the final feature list.

In standard ECFPs, each initial atom identifier is a 32 bit ($\approx$ ten digit) integer which contains the following information: 1. number of immediate neighbors who are heavy (non-hydrogen) atoms, 2. the valence minus the number of hydrogens, 3. the atomic number, 4. the atomic mass, 5. the atomic charge, 6. the number of attached hydrogens (both implicit and explicit), and 7. whether the atom is contained in at least one ring. What information is encoded in each atom identifier can be modified, and thus many variants of ECFPs exist.

E-state fingerprints (Hall & Kier, 1995)[7] The Estate fingerprint is based on electrotopological state (E-state) indices,[8] which encode information about associated functional group, graph topology, and the Kier-Hall electronegativity of each atom. Mathematically, unlike in ECFPs, the E-state indices for each atom are graph invariants, which means their value does not change when the labeling of nodes is permuted. The E-state fingerprint differs from traditional fingerprints as it is fixed length, containing a vector with counts of 79 atom types. Thus, it is more of a descriptor set than a fingerpint. We found that only 31 of the atom types were relevant to the energetic materials we studied. The E-state fingerprint can also be calculated as a real-valued vector which sums the E-state indices for each atom, however we found the predictive performance was exactly the same as with the count vector version.

Avalon fingerprints (Gedeck, et al., 2006)[9] Avalon fingerprints hash together a variety of information on atom pairs, atom triplets, graph topology, atomic number, bonding types, and bonding environments.

RDKit Fingerprint (Landrum, 2006[10]) The RDKit fingerprint is a simple fingerprinting algorithm which enumerates all paths/subgraphs up to a certain size, hashes them into bit vectors, and then OR's the bitvectors together into a fingerprint vector.

For two other RDKit fingerprints we tested, the extended reduced graph fingerprint (ErG),[11] and physio-chemical property fingerprints,[12] we were not able to vary the fingerprint length. (ErG is fixed length and physiochemical property fingerprints could only be used at the default length of 1024 due to constraints in RDKit). However both of these performed worse than the all others and are not shown on the plot. This is likely because both of these fingerprints were designed specifically with biological applications in mind.

ErG fingerprints (Stiefl, et al., 2006)[11] The extended reduced graph (ErG) fingerprint is generated by enumerating reduced graphs. Reduced graphs are a custom encoding scheme which retains chemically relevant information about graph substructures while throwing out superfluous information. ErG fingerprints contain information about graph topology, atomic charge, and H-bond donor and acceptor sites.

Physiochemical property fingerprints (Kearsley, et

al. 1997)[12] These are modification of atom-pair fingerprints were the atom types are determined by a triplet of information: binding property class, atomic *logP* contribution, and partial atomic charges.

[1] D. H. Wolpert, Neural Networks **5**, 241 (1992), ISSN 0893-6080.

[2] L. Breiman, Machine Learning **24**, 49 (1996), ISSN 1573-0565.

[3] R. E. Carhart, D. H. Smith, and R. Venkataraghavan, Journal of Chemical Information and Computer Sciences **25**, 64 (1985).

[4] R. Nilakantan, N. Bauman, J. S. Dixon, and R. Venkataraghavan, Journal of Chemical Information and Computer Sciences **27**, 82 (1987).

[5] D. Rogers and M. Hahn, J. Chem. Info. Mod. **50**, 742 (2010).

[6] H. L. Morgan, Journal of Chemical Documentation **5**, 107 (1965).

[7] L. H. Hall and L. B. Kier, Journal of Chemical Information and Computer Sciences **35**, 1039 (1995).

[8] L. B. Kier and L. H. Hall, Pharmaceutical Research **7**, 801 (1990).

[9] P. Gedeck, B. Rohde, and C. Bartels, J. Chem. Info. Mod. **46**, 1924 (2006).

[10] G. Landrum, *RDKit: Open-source cheminformatics*, `http://www.rdkit.org`.

[11] N. Stiefl, I. A. Watson, K. Baumann, and A. Zaliani, J. Chem. Info. Mod. **46**, 208 (2006).

[12] S. K. Kearsley, S. Sallamack, E. M. Fluder, J. D. Andose, R. T. Mosley, and R. P. Sheridan, Journal of Chemical Information and Computer Sciences **36**, 118 (1996).

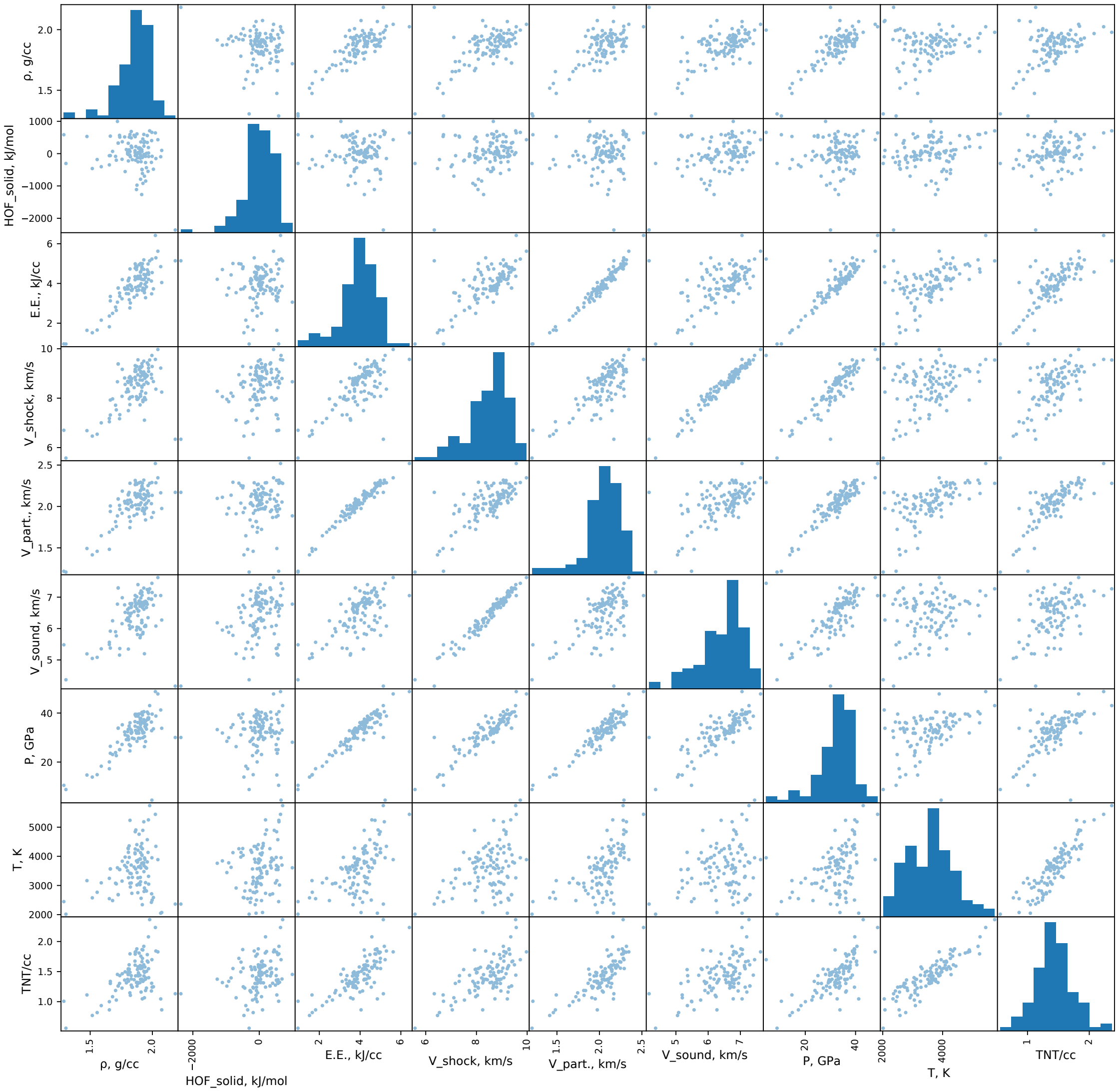

FIG. 1. Correlation plot visualization of the Huang & Massa property data.

name	$\mathrm{MAE}_{\mathrm{train}}$	$\mathrm{MAE}_{\mathrm{test}}$	$\mathrm{MAPE}_{\mathrm{test}}$	$\mathrm{RMSE}_{\mathrm{test}}$	R^2_{train}	R^2_{test}	r_{train}	r_{test}
Kernel Ridge	0.32 ± 0.02	0.35 ± 0.06	10.48	0.464	0.80	0.74	0.80	0.76
Bayesian Ridge	0.31 ± 0.01	0.36 ± 0.05	11.16	0.480	0.80	0.68	0.80	0.70
Elastic Net	0.31 ± 0.01	0.37 ± 0.05	11.13	0.485	0.80	0.68	0.80	0.72
Lasso	0.32 ± 0.01	0.38 ± 0.04	11.13	0.489	0.79	0.71	0.79	0.72
Ridge	0.32 ± 0.01	0.39 ± 0.05	11.44	0.500	0.79	0.66	0.80	0.73
Linear	0.30 ± 0.01	0.39 ± 0.02	11.52	0.510	0.81	0.65	0.81	0.68
Random Forest	0.19 ± 0.01	0.48 ± 0.06	14.48	0.631	0.92	0.49	0.94	0.53
Support Vector	0.18 ± 0.01	0.48 ± 0.10	17.33	0.671	0.89	0.45	0.93	0.50
Gradient Boosted Trees	0.04 ± 0.01	0.49 ± 0.04	14.45	0.628	0.99	0.43	1.00	0.49
k-Nearest Neighbors	0.35 ± 0.03	0.49 ± 0.09	15.67	0.668	0.78	0.44	0.79	0.52
Gaussian Process	0.00 ± 0.00	0.63 ± 0.15	23.45	0.889	1.00	0.04	1.00	0.15
Neural Network	0.76 ± 0.02	0.96 ± 0.10	28.03	1.250	-0.04	-0.92	0.29	0.19
Take the mean	0.68 ± 0.02	0.69 ± 0.08	24.91	0.935	0.00	-0.05	0.00	0.00

TABLE III. A complete comparison of all the models tested for explosive energy prediction with the sum over bonds featurization and 5-fold cross validation. One train-test split ($n_{\mathrm{train}}/n_{\mathrm{test}} = 5$) is shown for case, along with the mean average error and Pearson correlation r averaged over the 5 train-test splits. Gaussian process regression can improved with by implementing custom kernels (our result uses the default RBF kernel). We found neural network models could be found with more more extensive hyperparameter optimization, but still were worse than Kernel Ridge.

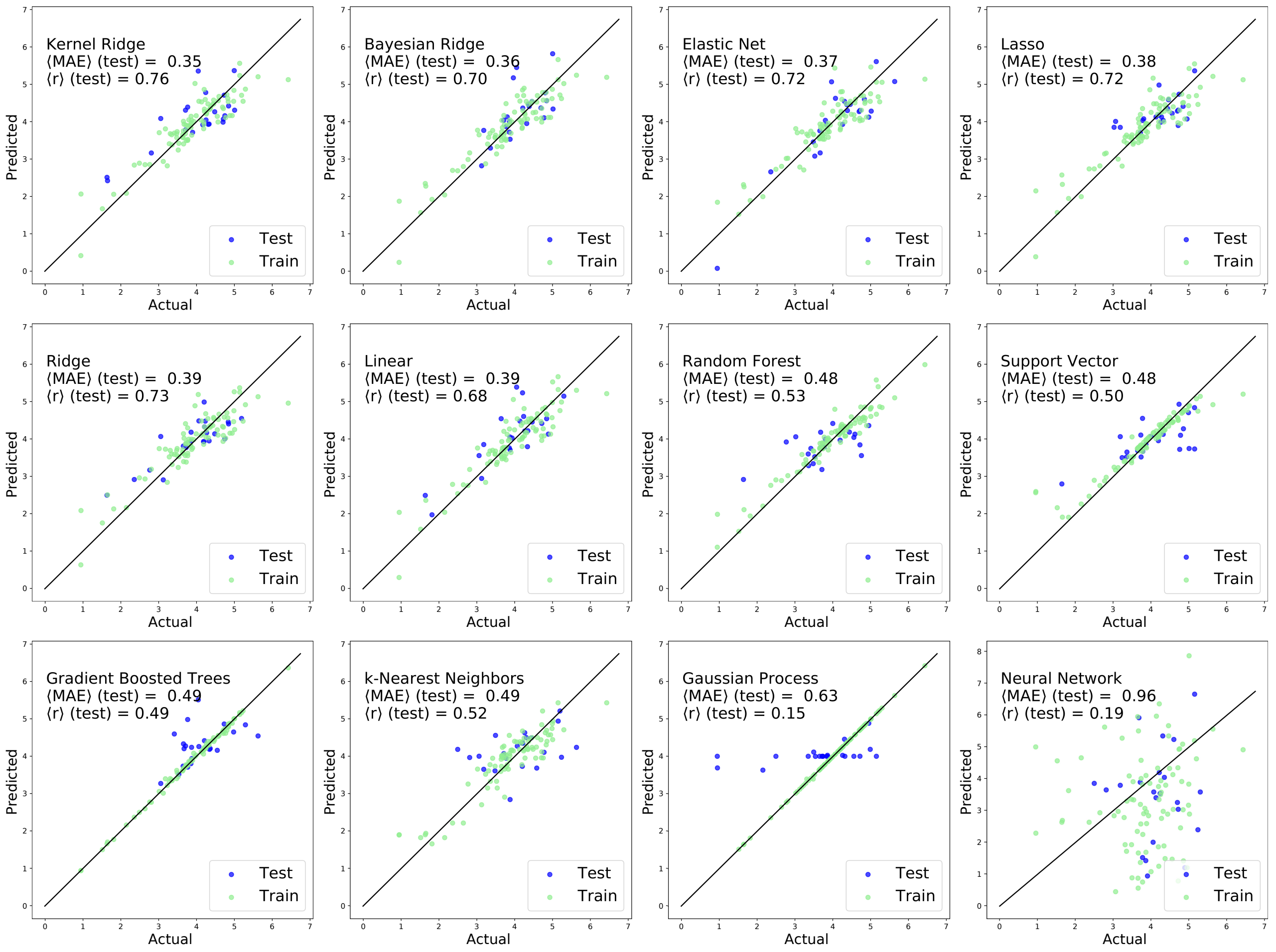

FIG. 2. A complete comparison of all the models tested for explosive energy prediction with the sum over bonds featurization and 5-fold cross validation.

		ρ, $\frac{\text{g}}{\text{cc}}$	ΔH_f^{s}, $\frac{\text{kJ}}{\text{mol}}$	E_{e}, $\frac{\text{kJ}}{\text{cc}}$	V_{s}, $\frac{\text{km}}{\text{s}}$	V_{p}, $\frac{\text{km}}{\text{s}}$	V_{snd}, $\frac{\text{km}}{\text{s}}$	P, GPa	T, K	$\frac{\text{TNT}_{\text{equiv}}}{\text{cc}}$
KRR	Estate	$0.10^{0.11}_{0.09}$	$261.02^{292.53}_{229.51}$	$0.63^{0.67}_{0.59}$	$0.48^{0.54}_{0.43}$	$0.13^{0.14}_{0.12}$	$0.41^{0.45}_{0.37}$	$4.95^{5.32}_{4.59}$	$500.19^{542.56}_{457.82}$	$0.18^{0.20}_{0.16}$
	CDS	$0.08^{0.09}_{0.07}$	$198.81^{211.87}_{185.76}$	$0.50^{0.55}_{0.46}$	$0.44^{0.49}_{0.39}$	$0.11^{0.12}_{0.10}$	$0.37^{0.41}_{0.34}$	$3.07^{3.38}_{2.76}$	$462.63^{513.95}_{411.32}$	$0.17^{0.19}_{0.16}$
	CM eigs	$0.09^{0.11}_{0.08}$	$288.41^{315.10}_{261.72}$	$0.67^{0.75}_{0.59}$	$0.67^{0.74}_{0.61}$	$0.18^{0.20}_{0.16}$	$0.61^{0.71}_{0.52}$	$5.67^{6.10}_{5.23}$	$600.08^{658.08}_{542.09}$	$0.22^{0.24}_{0.20}$
	Bag of Bonds	$0.06^{0.07}_{0.06}$	$166.66^{189.10}_{144.23}$	$0.47^{0.51}_{0.44}$	$0.33^{0.35}_{0.30}$	$0.11^{0.12}_{0.10}$	$0.29^{0.33}_{0.25}$	$3.38^{3.66}_{3.09}$	$478.93^{522.32}_{435.54}$	$0.18^{0.20}_{0.17}$
	Estate+CDS+SoB	$\mathbf{0.06}^{0.07}_{0.06}$	$\mathbf{71.40}^{79.69}_{63.12}$	$\mathbf{0.36}^{0.39}_{0.33}$	$0.32^{0.35}_{0.30}$	$0.10^{0.10}_{0.09}$	$0.29^{0.31}_{0.28}$	$\mathbf{2.76}^{2.98}_{2.55}$	$359.66^{396.00}_{323.31}$	$0.13^{0.15}_{0.12}$
	OB_{1600}	$0.07^{0.08}_{0.07}$	$368.32^{400.49}_{336.15}$	$0.48^{0.52}_{0.45}$	$0.53^{0.59}_{0.48}$	$0.18^{0.28}_{0.07}$	$0.50^{0.58}_{0.41}$	$3.43^{3.91}_{2.96}$	$704.57^{924.34}_{484.80}$	$0.22^{0.24}_{0.20}$
SVR	Estate	$0.09^{0.10}_{0.09}$	$207.78^{234.27}_{181.30}$	$0.60^{0.64}_{0.57}$	$0.45^{0.50}_{0.41}$	$0.13^{0.14}_{0.11}$	$0.35^{0.37}_{0.33}$	$4.41^{4.81}_{4.00}$	$476.06^{518.97}_{433.15}$	$0.17^{0.18}_{0.16}$
	CDS	$0.07^{0.08}_{0.07}$	$223.24^{257.71}_{188.77}$	$0.52^{0.56}_{0.49}$	$0.34^{0.37}_{0.31}$	$0.12^{0.13}_{0.11}$	$0.32^{0.35}_{0.29}$	$3.21^{3.42}_{3.01}$	$436.81^{475.64}_{397.99}$	$0.18^{0.19}_{0.16}$
	SoB	$0.06^{0.07}_{0.06}$	$130.78^{152.42}_{109.13}$	$0.40^{0.43}_{0.37}$	$\mathbf{0.31}^{0.33}_{0.29}$	$0.10^{0.11}_{0.09}$	$0.28^{0.30}_{0.26}$	$2.97^{3.16}_{2.78}$	$331.27^{364.66}_{297.88}$	$0.14^{0.16}_{0.13}$
	CM eigs	$0.08^{0.09}_{0.07}$	$288.41^{326.75}_{250.08}$	$0.55^{0.58}_{0.52}$	$0.60^{0.65}_{0.55}$	$0.15^{0.16}_{0.14}$	$0.53^{0.58}_{0.48}$	$4.54^{4.94}_{4.13}$	$584.44^{624.58}_{544.29}$	$0.21^{0.23}_{0.20}$
	Bag of Bonds	$0.07^{0.08}_{0.07}$	$159.24^{188.03}_{130.45}$	$0.47^{0.50}_{0.44}$	$0.35^{0.37}_{0.32}$	$0.12^{0.12}_{0.11}$	$0.28^{0.31}_{0.26}$	$3.34^{3.53}_{3.15}$	$385.59^{419.20}_{351.97}$	$0.18^{0.19}_{0.17}$
	Estate+CDS+SoB	$0.06^{0.06}_{0.05}$	$129.89^{148.84}_{110.93}$	$0.37^{0.40}_{0.35}$	$0.34^{0.38}_{0.31}$	$0.10^{0.10}_{0.09}$	$0.28^{0.31}_{0.26}$	$\mathbf{2.73}^{2.91}_{2.55}$	$353.18^{379.95}_{326.41}$	$0.13^{0.15}_{0.12}$
	OB_{1600}	$0.07^{0.08}_{0.06}$	$337.94^{373.47}_{302.41}$	$0.52^{0.56}_{0.47}$	$0.44^{0.47}_{0.41}$	$0.12^{0.13}_{0.11}$	$0.41^{0.43}_{0.38}$	$3.78^{4.02}_{3.54}$	$546.42^{590.40}_{502.44}$	$0.19^{0.20}_{0.18}$
Ridge	Estate	$0.09^{0.10}_{0.09}$	$269.11^{302.51}_{235.70}$	$0.58^{0.63}_{0.53}$	$0.57^{0.60}_{0.53}$	$0.14^{0.15}_{0.13}$	$0.45^{0.48}_{0.42}$	$4.71^{5.01}_{4.41}$	$491.21^{534.08}_{448.34}$	$0.19^{0.20}_{0.17}$
	CDS	$0.07^{0.07}_{0.06}$	$193.19^{207.17}_{179.21}$	$0.43^{0.47}_{0.40}$	$0.39^{0.43}_{0.35}$	$0.11^{0.12}_{0.10}$	$0.33^{0.35}_{0.31}$	$3.23^{3.56}_{2.91}$	$438.27^{467.92}_{408.61}$	$0.17^{0.18}_{0.15}$
	SoB	$\mathbf{0.06}^{0.06}_{0.05}$	$82.00^{88.76}_{75.24}$	$\mathbf{0.37}^{0.40}_{0.34}$	$0.32^{0.34}_{0.30}$	$0.10^{0.11}_{0.09}$	$0.29^{0.31}_{0.27}$	$3.01^{3.20}_{2.82}$	$\mathbf{327.43}^{358.22}_{296.65}$	$\mathbf{0.11}^{0.12}_{0.11}$
	CM eigs	$0.09^{0.10}_{0.08}$	$355.12^{396.42}_{313.81}$	$0.79^{0.92}_{0.65}$	$0.60^{0.64}_{0.57}$	$0.16^{0.19}_{0.13}$	$0.55^{0.59}_{0.51}$	$5.82^{6.37}_{5.26}$	$590.69^{635.69}_{545.69}$	$0.19^{0.20}_{0.18}$
	Bag of Bonds	$0.06^{0.07}_{0.06}$	$163.76^{185.79}_{141.74}$	$0.48^{0.51}_{0.45}$	$0.32^{0.35}_{0.29}$	$0.11^{0.12}_{0.10}$	$0.31^{0.33}_{0.29}$	$3.37^{3.67}_{3.07}$	$472.93^{512.03}_{433.83}$	$0.19^{0.21}_{0.18}$
	Estate+CDS+SoB	$0.06^{0.06}_{0.06}$	$77.31^{84.13}_{70.48}$	$0.39^{0.41}_{0.36}$	$0.32^{0.33}_{0.31}$	$0.10^{0.11}_{0.09}$	$0.28^{0.31}_{0.25}$	$2.78^{2.95}_{2.60}$	$383.07^{415.60}_{350.54}$	$0.13^{0.14}_{0.12}$
	OB_{1600}	$0.06^{0.07}_{0.06}$	$362.89^{396.80}_{328.98}$	$0.49^{0.54}_{0.45}$	$0.51^{0.54}_{0.47}$	$0.11^{0.12}_{0.10}$	$0.46^{0.49}_{0.43}$	$3.39^{3.65}_{3.12}$	$545.27^{575.00}_{515.54}$	$0.19^{0.20}_{0.18}$
RF	Estate	$0.09^{0.10}_{0.09}$	$252.74^{291.84}_{213.65}$	$0.59^{0.64}_{0.54}$	$0.50^{0.54}_{0.46}$	$0.14^{0.15}_{0.13}$	$0.39^{0.42}_{0.36}$	$4.09^{4.38}_{3.80}$	$488.98^{534.33}_{443.63}$	$0.19^{0.21}_{0.18}$
	CDS	$0.07^{0.08}_{0.07}$	$241.67^{265.41}_{217.93}$	$0.46^{0.50}_{0.43}$	$0.36^{0.39}_{0.33}$	$0.11^{0.12}_{0.10}$	$0.29^{0.32}_{0.27}$	$3.34^{3.57}_{3.11}$	$435.77^{475.37}_{396.17}$	$0.16^{0.18}_{0.15}$
	SoB	$0.07^{0.08}_{0.07}$	$136.91^{157.28}_{116.55}$	$0.48^{0.51}_{0.45}$	$0.40^{0.44}_{0.37}$	$0.12^{0.13}_{0.10}$	$0.30^{0.33}_{0.28}$	$3.47^{3.74}_{3.21}$	$417.46^{456.54}_{378.39}$	$0.15^{0.16}_{0.13}$
	CM eigs	$0.09^{0.10}_{0.08}$	$286.89^{311.55}_{262.23}$	$0.67^{0.72}_{0.61}$	$0.62^{0.66}_{0.58}$	$0.15^{0.17}_{0.14}$	$0.51^{0.56}_{0.47}$	$5.52^{6.02}_{5.02}$	$512.22^{550.43}_{474.00}$	$0.20^{0.22}_{0.18}$
	Bag of Bonds	$0.07^{0.07}_{0.06}$	$172.41^{194.19}_{150.63}$	$0.46^{0.50}_{0.43}$	$0.36^{0.39}_{0.33}$	$0.10^{0.11}_{0.10}$	$0.29^{0.32}_{0.25}$	$3.10^{3.29}_{2.92}$	$418.35^{449.87}_{386.83}$	$0.16^{0.18}_{0.15}$
	Estate+CDS+SoB	$0.07^{0.08}_{0.07}$	$144.18^{165.03}_{123.34}$	$0.43^{0.47}_{0.39}$	$0.34^{0.36}_{0.31}$	$\mathbf{0.09}^{0.10}_{0.08}$	$\mathbf{0.26}^{0.29}_{0.24}$	$3.11^{3.39}_{2.82}$	$401.27^{441.52}_{361.01}$	$0.15^{0.16}_{0.14}$
	OB_{1600}	$0.08^{0.08}_{0.07}$	$443.56^{473.61}_{413.51}$	$0.48^{0.53}_{0.43}$	$0.48^{0.51}_{0.46}$	$0.12^{0.13}_{0.11}$	$0.45^{0.48}_{0.42}$	$3.58^{3.95}_{3.21}$	$611.27^{654.07}_{568.47}$	$0.21^{0.23}_{0.19}$
kNN	Estate	$0.08^{0.09}_{0.08}$	$236.55^{266.19}_{206.91}$	$0.61^{0.64}_{0.57}$	$0.49^{0.52}_{0.46}$	$0.15^{0.16}_{0.14}$	$0.41^{0.43}_{0.38}$	$4.30^{4.62}_{3.99}$	$563.89^{609.25}_{518.53}$	$0.20^{0.21}_{0.18}$
	CDS	$0.07^{0.08}_{0.07}$	$242.99^{271.36}_{214.62}$	$0.55^{0.59}_{0.51}$	$0.39^{0.42}_{0.36}$	$0.13^{0.14}_{0.12}$	$0.33^{0.35}_{0.30}$	$3.56^{3.75}_{3.38}$	$478.50^{508.42}_{448.57}$	$0.18^{0.19}_{0.17}$
	SoB	$0.08^{0.08}_{0.07}$	$184.43^{209.57}_{159.29}$	$0.54^{0.58}_{0.50}$	$0.44^{0.48}_{0.39}$	$0.12^{0.14}_{0.11}$	$0.36^{0.38}_{0.33}$	$3.65^{3.93}_{3.38}$	$427.20^{461.44}_{392.96}$	$0.17^{0.19}_{0.16}$
	CM eigs	$0.10^{0.11}_{0.09}$	$343.48^{368.70}_{318.27}$	$0.62^{0.67}_{0.56}$	$0.67^{0.72}_{0.62}$	$0.15^{0.16}_{0.13}$	$0.51^{0.54}_{0.49}$	$5.52^{5.84}_{5.21}$	$570.55^{604.71}_{536.39}$	$0.22^{0.24}_{0.21}$
	Bag of Bonds	$0.08^{0.09}_{0.07}$	$238.05^{258.83}_{217.27}$	$0.53^{0.57}_{0.49}$	$0.40^{0.44}_{0.37}$	$0.11^{0.12}_{0.10}$	$0.32^{0.35}_{0.29}$	$3.58^{3.88}_{3.29}$	$515.25^{546.78}_{483.71}$	$0.19^{0.20}_{0.18}$
	Estate+CDS+SoB	$0.08^{0.08}_{0.07}$	$171.65^{195.35}_{147.94}$	$0.54^{0.59}_{0.49}$	$0.43^{0.46}_{0.40}$	$0.12^{0.13}_{0.11}$	$0.35^{0.37}_{0.33}$	$3.57^{3.76}_{3.37}$	$442.14^{476.56}_{407.73}$	$0.17^{0.18}_{0.16}$
	OB_{1600}	$0.08^{0.08}_{0.07}$	$333.95^{359.50}_{308.41}$	$0.49^{0.53}_{0.44}$	$0.50^{0.53}_{0.46}$	$0.11^{0.12}_{0.10}$	$0.45^{0.49}_{0.42}$	$3.45^{3.73}_{3.17}$	$515.62^{554.44}_{476.80}$	$0.19^{0.21}_{0.17}$

TABLE IV. Average mean absolute errors in the test sets for different combinations of target property, model and featurization. Hyperparameter optimization and 20-fold shuffle split with a train-test ratio of 4:1 was used. 95% confidence intervals for the mean errors were calculated using the standard statistical method with the corresponding t value.

		ρ, $\frac{\text{g}}{\text{cc}}$	ΔH_f^{s}, $\frac{\text{kJ}}{\text{mol}}$	E_{e}, $\frac{\text{kJ}}{\text{cc}}$	V_{s}, $\frac{\text{km}}{\text{s}}$	V_{p}, $\frac{\text{km}}{\text{s}}$	V_{snd}, $\frac{\text{km}}{\text{s}}$	P (GPa)	T (K)	$\frac{\text{TNT}_{\text{equiv}}}{\text{cc}}$	avg
KR	Estate	0.69	0.39	0.72	0.53	0.58	0.56	0.55	0.51	0.60	0.57
	CDS	0.57	0.63	0.56	0.57	0.44	0.50	0.45	0.50	0.42	0.52
	SoB	0.70	0.95	0.72	0.70	0.67	0.73	0.67	0.61	0.70	**0.72**
	CM eigs	0.40	0.33	0.52	0.25	0.51	0.20	0.33	0.21	0.34	0.34
	Bag of Bonds	0.61	0.78	0.72	0.73	0.56	0.75	0.61	0.46	0.49	0.63
	Estate+CDS+SoB	0.74	0.94	0.78	0.70	0.65	0.71	0.65	0.50	0.65	0.70
SVR	Estate	0.55	0.57	0.56	0.70	0.70	0.61	0.60	0.52	0.53	0.59
	CDS	0.39	0.06	0.38	0.51	0.37	0.48	0.46	0.34	0.31	0.37
	SoB	0.38	0.61	0.60	0.62	0.59	0.58	0.57	0.58	0.57	0.57
	CM eigs	0.02	0.05	0.03	0.05	0.03	0.05	0.03	0.06	0.11	0.05
	Bag of Bonds	0.04	0.12	0.04	0.14	0.10	0.15	0.10	0.14	0.10	0.10
	Estate+CDS+SoB	0.32	0.58	0.46	0.56	0.43	0.54	0.47	0.56	0.58	0.50
Ridge	Estate	0.69	0.28	0.69	0.56	0.66	0.44	0.61	0.45	0.51	0.54
	CDS	0.64	0.63	0.60	0.40	0.46	0.45	0.40	0.50	0.49	0.51
	SoB	0.71	0.93	0.69	0.78	0.63	0.74	0.63	0.68	0.71	**0.72**
	CM eigs	0.46	0.20	0.43	0.13	0.32	0.12	0.30	0.19	0.22	0.26
	Bag of Bonds	0.47	0.74	0.63	0.73	0.53	0.75	0.57	0.36	0.39	0.58
	Estate+CDS+SoB	0.70	0.94	0.79	0.76	0.69	0.69	0.65	0.60	0.70	**0.72**
Lasso	Estate	0.58	0.34	0.69	0.50	0.61	0.49	0.44	0.39	0.41	0.49
	CDS	0.54	0.68	0.55	0.41	0.48	0.41	0.43	0.53	0.48	0.50
	SoB	0.62	0.94	0.73	0.77	0.69	0.78	0.60	0.63	0.63	0.71
	CM eigs	0.41	0.26	0.38	0.12	0.31	0.13	0.21	0.24	0.23	0.25
	Bag of Bonds	0.43	0.66	0.50	0.75	0.55	0.75	0.45	0.33	0.39	0.53
	Estate+CDS+SoB	0.71	0.95	0.73	0.75	0.69	0.78	0.62	0.54	0.67	**0.72**
BR	Estate	0.66	0.38	0.70	0.54	0.64	0.51	0.59	0.36	0.48	0.54
	CDS	0.62	0.67	0.57	0.47	0.47	0.39	0.40	0.46	0.42	0.50
	SoB	0.67	0.93	0.73	0.73	0.75	0.77	0.59	0.55	0.70	0.71
	CM eigs	0.42	0.18	0.37	0.10	0.34	0.26	0.18	0.21	0.21	0.25
	Bag of Bonds	0.55	0.78	0.55	0.72	0.48	0.72	0.57	0.27	0.41	0.56
	Estate+CDS+SoB	0.77	0.95	0.74	0.77	0.66	0.75	0.65	0.59	0.60	**0.72**
GBoost	Estate	0.43	0.39	0.52	0.43	0.53	0.54	0.39	0.37	0.35	0.44
	CDS	0.49	0.47	0.43	0.48	0.46	0.56	0.39	0.49	0.43	0.47
	SoB	0.60	0.78	0.62	0.61	0.60	0.56	0.45	0.60	0.40	0.58
	CM eigs	0.23	0.42	0.13	0.15	0.07	0.02	0.10	0.27	0.27	0.18
	Bag of Bonds	0.66	0.75	0.58	0.73	0.56	0.70	0.49	0.55	0.56	0.62
	Estate+CDS+SoB	0.64	0.81	0.61	0.64	0.60	0.63	0.43	0.59	0.52	0.61
RF	Estate	0.47	0.41	0.39	0.50	0.62	0.56	0.42	0.35	0.41	0.46
	CDS	0.56	0.44	0.42	0.44	0.41	0.46	0.41	0.44	0.42	0.45
	SoB	0.56	0.79	0.55	0.63	0.55	0.60	0.41	0.52	0.41	0.56
	CM eigs	0.22	0.32	0.22	0.18	0.23	0.10	0.19	0.30	0.29	0.23
	Bag of Bonds	0.68	0.72	0.63	0.67	0.62	0.71	0.47	0.51	0.46	0.61
	Estate+CDS+SoB	0.63	0.79	0.64	0.62	0.56	0.68	0.53	0.52	0.49	0.60
kNN	Estate	0.42	0.43	0.46	0.52	0.44	0.47	0.49	0.36	0.39	0.44
	CDS	0.47	0.06	0.42	0.28	0.38	0.28	0.38	0.42	0.41	0.35
	SoB	0.49	0.65	0.46	0.62	0.46	0.58	0.46	0.48	0.53	0.53
	CM eigs	0.20	0.26	0.22	0.03	0.16	0.13	0.07	0.14	0.18	0.16
	Bag of Bonds	0.41	0.34	0.53	0.70	0.56	0.65	0.43	0.32	0.36	0.48
	Estate+CDS+SoB	0.54	0.50	0.55	0.58	0.44	0.54	0.58	0.40	0.45	0.51

TABLE V. Pearson correlation values in the test set for different combinations of target property, model and featurization. Hyperparameter optimization and 5-fold cross validation was used here.

		ρ, $\frac{g}{cc}$	ΔH^{s}_{f}, $\frac{kJ}{mol}$	E_e, $\frac{kJ}{cc}$	V_s, $\frac{km}{s}$	V_p, $\frac{km}{s}$	V_{snd}, $\frac{km}{s}$	P, GPa	T, K	$\frac{TNT_{equiv}}{cc}$
KR	CDS	3.97	108.13	16.22	5.38	6.95	5.99	13.57	12.69	12.95
	SoB	2.97	59.83	11.11	3.60	4.96	4.04	11.07	13.13	10.27
SVR	CDS	3.55	107.53	15.24	4.55	6.35	5.22	12.09	11.97	14.88
	SoB	3.31	67.36	11.21	4.17	5.21	4.55	9.56	10.68	8.33
Ridge	CDS	3.85	127.68	16.80	6.13	7.13	6.80	14.87	13.10	13.44
	SoB	3.24	70.76	11.98	3.60	4.85	4.41	10.25	10.60	8.52
mean	CDS	5.67	106.41	24.78	8.17	8.46	8.91	21.56	18.40	17.60
	SoB	5.70	113.62	24.55	7.98	8.62	8.87	21.45	18.58	17.60

TABLE VI. Mean absolute percentage errors for some of the models and two of the best featurization. Note that these numbers are often inflated by the presence of small target values, and may not accurately reflect model accuracy.

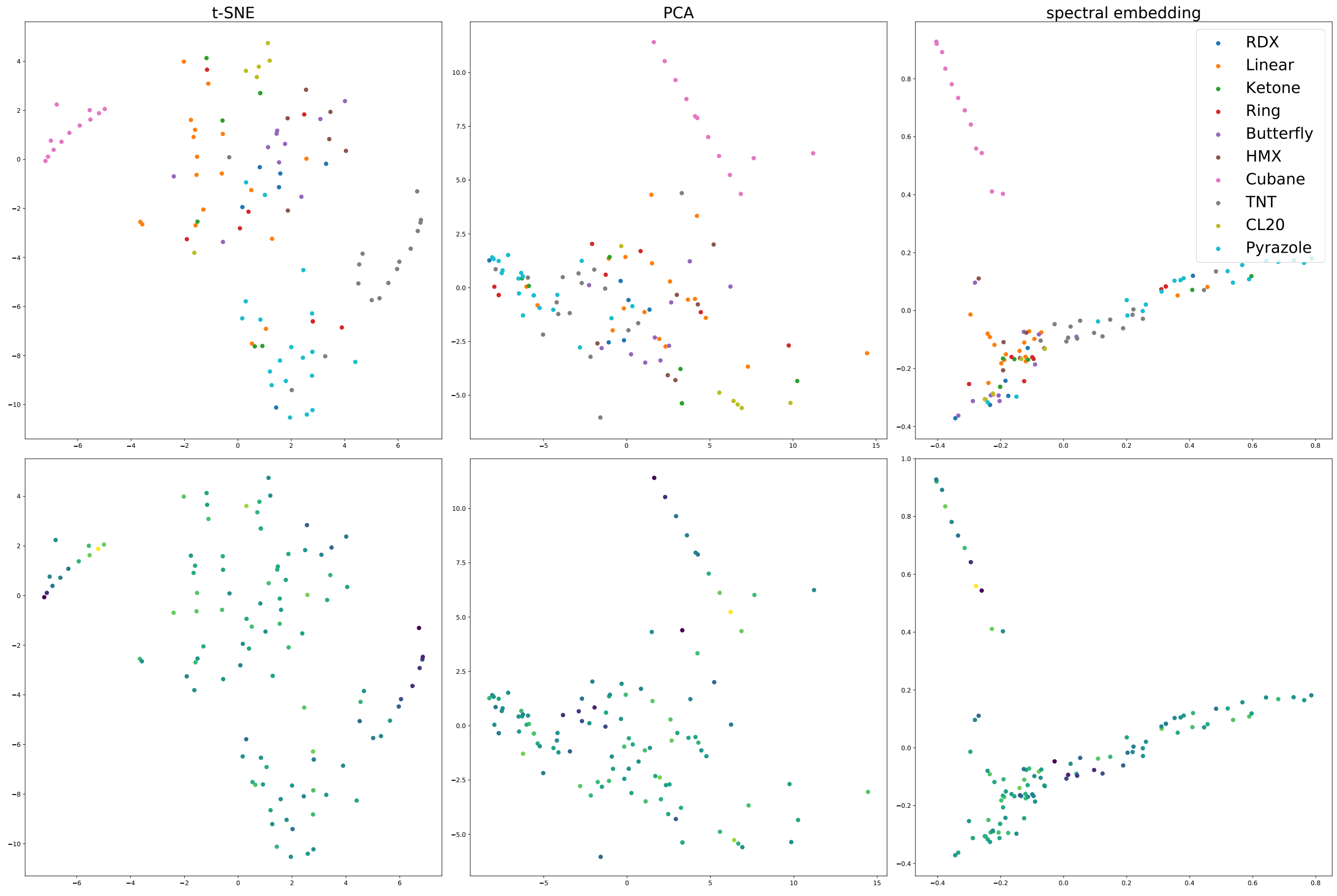

FIG. 3. Two dimensional embeddings of the Huang & Massa data using the sum over bonds featurization and three different embedding techniques - t-SNE, PCA, & spectral embedding. The upper figures show the different chemical groups while the bottom figures show the explosive energy (dark = low, light = high).
